# Symptom presentation, perceived causes, and help-seeking practices among adults receiving depression or anxiety care in Nepal: A qualitative study

**DOI:** 10.1371/journal.pone.0347605

**Published:** 2026-05-08

**Authors:** Nagendra P. Luitel, Kriti Pudasaini, Bishnu Lamichhane, Kamal Gautam, Mark J.D. Jordans

**Affiliations:** 1 Research Department, Transcultural Psychosocial Organization Nepal (TPO Nepal), Baluwatar, Kathmandu, Nepal; 2 Center for Global Mental Health Equity, Department of Psychiatry and Behavioural Health, The George Washington University, Washington, District of Columbia, United States of America; 3 Department of Global Public Health, Karolinska Institutet, Stockholm, Sweden; 4 Centre for Global Mental Health, Health Service and Population Research Department, Institute of Psychiatry, Psychology and Neuroscience, King’s College London, London, United Kingdom; Group for Technical Assistance / Asian College for Advance Studies, Purbanchal University, NEPAL

## Abstract

Depression and anxiety are prevalent worldwide, yet fewer than 20% of individuals in low- and middle-income countries receive appropriate care. Cultural norms play a significant role in how symptoms are expressed and how individuals seek help. In Nepal, traditional healers are often preferred over mental health specialists. This qualitative study explored how symptom presentation and perceived causes impact treatment-seeking behaviour among adults receiving care for depression or anxiety. Twenty-four participants (13 with depression, 9 with anxiety, and 2 with both conditions) were recruited from Jhapa, Chitwan, and Kailali districts through primary healthcare providers, psychosocial counselors, and mental health specialists. Individual interviews were conducted using the adapted McGill Illness Narrative Interview (MINI) to explore symptom experiences, illness narratives, perceived causes, and help-seeking patterns. Thematic analysis of the data was done using NVIVO software. Participants reported a range of emotional, cognitive, and physical symptoms including fear, anxiety, restlessness, irritability, sadness, and hopelessness. Somatic complaints like headaches, fatigue, and gastrointestinal issues were often interpreted as consequences of psychological stress. Some participants described dissociative experiences, such as detachment, amnesia, or perceptual distortions, leading to panic or self-harm. Stressors mentioned included financial hardships, bereavement, family conflicts, trauma, and culturally ingrained fears. Most participants initially sought help from biomedical providers like private clinics, hospitals, health centers, or health camps with some also consulting traditional healers. Education and caste played a significant role in treatment choices, with individuals with higher education and from higher castes more likely to seek biomedical care first. Gender and age had minimal impact. The study underscores the importance of culturally sensitive, community-based mental health programs to reduce stigma and ensure equitable access to care for depression and anxiety in Nepal.

## Introduction

Depression and anxiety are prevalent mental health conditions globally, affecting more than 322 million [1], and 374 million individuals [2], respectively. Factors such as low education levels, poor health, chronic stress, low self-esteem, unemployment, poverty, social isolation, and adverse life events increase the risk of depression and anxiety [3–6]. These conditions have a significant impact on individuals, families, and communities, affecting their quality of life [7,8]. Effective treatments are available through a task-sharing approach in the community [9,10], but individuals with depression or anxiety often do not seek help and may try to manage the condition on their own. In low- and middle-income countries (LMICs), less than 20% of those affected receive treatment, and of those who do, only 2 in 5 receive minimally adequate treatment [11,12]. Timely and appropriate treatment of these conditions can reduce the health burden and improve public health outcomes [13,14]. Challenges persist in detecting and addressing these conditions when integrating mental health services into primary health systems in LMICs, with fewer than one in ten individuals being accurately diagnosed by primary healthcare providers [15].

Symptom expression and cultural influences play a significant role in seeking help for depression or anxiety. Cultural norms determine what symptoms are perceived as “normal” or “abnormal,” how they are expressed, and the type of support systems deemed suitable [16]. For instance, individuals with depression or anxiety often manifest their suffering through physical complaints such as headaches, chest tightness, or numbness, attributing the cause to failures in meeting social obligations, gender roles, and spiritual imbalances [17]. Stigma associated with mental illness is influenced by cultural beliefs, attitudes, and values, varying across cultures. In Nepal, the term “heart-mind” problem is socially accepted, while the term “brain-mind” is highly stigmatized [18]. Some cultures prioritize physical symptoms like headaches or stomach aches as signs of distress, while others emphasize emotional symptoms such as sadness or anxiety [19,20]. Research indicates that men are often more hesitant to seek help for mental health issues than women [21], contributing to higher suicide rates among men [22,23]. Understanding these cultural differences is crucial for creating accurate diagnostic and treatment guidelines for depression and anxiety.

Research in Nepal on help-seeking behavior for mental health care is limited, but existing studies reveal a complex pattern involving various providers, including traditional healers and mental health specialists [24–26]. Only 17% of individuals with mental health conditions seek help directly from mental health specialists, while 28.2% seek assistance from traditional or religious faith healers [24,26]. There is a lack of research on how perceived causes and symptom presentation impact access to and continuation of treatment. To address this gap, we conducted a qualitative study with individuals currently undergoing treatment for depression or anxiety from healthcare professionals. The study aimed to explore how types of symptoms experienced and perceived causes of illness affect help-seeking practices.

## Materials and methods

### Setting

The study took place in Nepal, a country in South Asia with a population of 29.1 million. Nepal has a federal government system comprising 7 provinces and 77 districts. The study focused on Jhapa, Chitwan, and Kailali districts, which are known for their diverse populations and access to mental health services. Nepal’s healthcare system includes public, private, and NGO sectors, with a limited number of mental health professionals. Traditional healers continue to play a significant role, particularly in rural areas. While mental health services are more concentrated in urban areas, these districts have primary healthcare providers trained in World Health Organization’s mental health gap action program intervention guide (mhGAP-IG) along with some resources for mental health care.

### Study design

The study used a qualitative design to explore participants’ perspectives on the causes of depression or anxiety, how symptoms manifest, and the factors influencing seeking help from different providers. Qualitative research allows for a comprehensive examination of a topic with limited existing research and encourages participants to contribute to new knowledge [27]. Individual interviews (IDI) were conducted to delve into personal experiences and challenges, as they provide depth, flexibility, and the opportunity to establish a confidential and trusting relationship.

### Participants, sample size and sampling process

The study involved adults undergoing treatment for depression or anxiety from trained primary healthcare providers, psychosocial counselors or mental health specialists. To ensure the trustworthiness of the qualitative study, we adhered to Lincoln and Guba’s criteria [28], which included conducting the study in various districts to ensure diversity in terms of caste/ethnicity, cultural practices, and geography. We collaborated with different service providers for participant recruitment, employed experienced interviewers of the same gender as the participants, involved multiple individuals in data analysis, and employed thematic and framework analysis methods. A total of 24 participants were enrolled, with 13 diagnosed with depression, 9 with anxiety disorder, and 2 with both conditions. Seven participants were from Kailali, four from Chitwan, and thirteen from Jhapa district. Recruitment sources included primary healthcare providers (7), psychosocial counselors (7), and psychiatrists (10). Participants were purposively selected based on pre-defined criteria which included being 18 years or older, currently receiving mental health treatment from primary healthcare providers, psychosocial counselors, or mental health specialists, capable of providing informed consent, and proficient in Nepali.

### Recruitment and interview process

Two researchers with university degree and several years of experience in qualitative research conducted interviews, pairing interviewers and participants by gender to reduce potential bias. The researchers received two weeks of training, which covered qualitative interviewing techniques, inclusion and exclusion criteria, and an orientation to the interview guides, with particular emphasis on using non-stigmatized terminologies and maintaining confidentiality throughout the interview process and data handling. The first author supervised the interviewers by reviewing audio recordings and providing feedback regularly. Trained interviewers visited participants at homes, explained the study’s objectives, benefits, and risks, and obtained written consent. Interviews were conducted either at home or in a confidential space in the outpatient department at a health center. All interviews were audio recorded in participants’ own words and lasted 45–60 minutes, depending on the depth of information shared. The interviews took place between January 20, 2023 and July 4, 2023.

### Interview guides

We adapted the McGill Illness Narrative Interview (MINI), a semi-structured interview protocol commonly used in mental health research, to collect detailed narratives on symptom experiences, illness narratives, perceived causes of illness and help-seeking behaviors [29,30]. The interview guide comprised five sections: history and understanding of the illness, effects and perceived causes, experience of stigma and discrimination, treatment pathways, and barriers and facilitators to treatment. Prior to data collection, the interview guide underwent pilot testing with a small group of participants. This paper focuses on data related to symptoms experienced, perceived causes and treatment pathways.

### Data management and analysis

All interviews were audio-recorded, transcribed verbatim into Nepali by the interviewers immediately after each interview, and later translated into English for analysis. The first author reviewed all transcripts against the audio-recordings to ensure accuracy and preserve culturally embedded meaning. Idioms of distress were translated to achieve conceptual rather than literal equivalence, with consultation from an experienced psychiatrist. Two researchers who were involved in coding and data entry independently reviewed all transcripts for initial familiarization.

Data were analyzed thematically using NVivo (version 20). The process began with open-coding of two transcripts to generate initial codes. These initial codes guided the development of a structured coding framework and codebook, which included parent and child nodes with definitions and quotations. Parent nodes covered major analytic domains, such as somatic, emotional, cognitive, behavioural, and cultural expressions of distress, perceived causes, and treatment-seeking pathways. Child nodes represented specific idiomatic expression; translated into English descriptors while retaining the original Nepali terms in italics for nuance. Multi-coding was employed when excerpts related to multiple domains (S1 Table).

Coding consistency and thematic saturation were monitored throughout the analysis. Inter-coder agreement was assessed by having two researchers independently code six additional transcripts. Discrepancies were resolved through discussion with the first author and a psychiatrist to ensure clinical and cultural validity. Inter-coder reliability was confirmed before proceeding with coding of the remaining transcripts (Cohen’s kappa = 0.71). Thematic saturation was reached when new codes or concepts stopped emerging, and thematic patterns stabilized across cases. After cross-case thematic analysis, individual interviews were examined holistically to explore co-occurring symptoms and help-seeking.

### Reflexivity

The authors’ backgrounds and expertise in clinical psychology, psychiatry, and global mental health may have shaped the framing and interpretation of the data, potentially highlighting biomedical and service-focused perspectives. To counteract this potential bias, the team engaged in ongoing reflexive practice, challenging assumptions, collaboratively reviewing coding, and addressing both clinical and cultural biases. Input from local experts and regular interdisciplinary discussions supported culturally sensitive interpretations and helped maintain fidelity to authentic local expressions of distress and help-seeking.

### Ethical Statement

This study was conducted in compliance with the Declaration of Helsinki and received ethical approval from the Nepal Health Research Council (NHRC) (Registration number: 527/2022 P). Each participant signed a written informed consent before enrolling in the study. Only those participants who voluntarily agreed to participate were included in the study.

## Results and discussion

Table 1 provides an overview of the sociodemographic characteristics, symptoms, perceived causes, and help-seeking practices of 24 participants undergoing treatment for depression or anxiety. The participants, aged 18–60, were mostly married (N = 20) and predominantly female (N = 15). They had varied educational backgrounds, with most completing basic education (N = 10), followed by secondary level (N = 9), higher education (N = 3), and some with no formal education (N = 2). Ethnically, participants were primarily Brahmin (N = 7), Chhetri (N = 6), Dalit (N = 7), and Janajati (N = 4). Their occupations included agriculture (N = 5), business (N = 5), homemaking (N = 4), foreign employment (N = 3), and other occupations (N = 7).

**Table 1:** Summary of the results.

ID	Socio demographic information	Symptoms experienced	Perceived Causes	Places/providers visited to seek care
P19	59-year male, married Janajati, grade-8, occupation- agriculture	Fearfulness, dissociative spells (fighting shadows, fighting ghosts, freezing sensation), difficulty in breathing, neck stiffness, and thoughts of self-harm	- Possessed by a ghost - Financial difficulties	•Traditional Healer (× 2) → Private Clinic (× 1) → Private Hospital (× 2) → Traditional Healer (× 1) → Private Hospital (× 1) → Health Camp (× 1)
P9	60-year-old married Chhetri male, education bachelor’s degree, occupation retired teacher	Fearfulness, social withdrawal, sadness, excessive thoughts and worries, startled response, feeling lonely, forgetfulness, low self-esteem, insomnia, vacant stares *(tolaune),* palpitations, and loss of interest	- Bereavement (death of son in law) - Lack of support from family - Financial difficulty	•Private Clinic (× 1) → Government Hospital (× 1)
P7	25-year-old unmarried Chhetri male, education grade 12, occupation foreign employment	Social withdrawal, loss of interest, fatigue, irritability, low self-confidence, insomnia, appetite loss, hopelessness, panic attacks, restlessness, feeling suffocated, palpitations, burning sensation (head), difficulty concentrating, increased blood pressure	•- Bereavement (death of son-in-law) •- Family illness (daughter-in-law’s cancer and depression) - Emotional distance (son abroad)	•Government Hospital (× 2)
P22	30-year-old married Brahmin male, education Grade 12, occupation business	Palpitations, irritability, fearfulness, restlessness, hopelessness *(niraash),* anhedonia, fatigue, weight gain, social withdrawal	•- COVID-19 infection for long time •- Financial stress (repaying debt) •- Children staying abroad - Physical health issues	Private Hospital → Government Hospital → Private Clinic →Private hospital (× 3)
P23	45-year-old married Chhetri female, education Grade 6, occupation business	Fear, restlessness, low self-confidence, fatigue, breathing difficulty, numbness of limb, insomnia, excessive sweating, nervousness, low self-esteem, palpitations, chest heaviness	•- Financial stress (repaying debt)	Traditional Healer (× 1) → Private Clinic (× 1) → Private Hospital (× 1) → Traditional Healer (× 1) Private Hospital (× 1)
P6	35-year-old married Dalit male, education Grade 10, occupation foreign employment	Insomnia, racing thoughts, chest pain, startle response, weight loss, palpitations, negative thoughts, fear, restlessness, social isolation, headache, shortness of breath, burning sensation (eyes), irritability, and dryness of mouth	- Bereavement (father’s death) - Marital conflict - Sole financial/household responsibility	Private Clinic (× 1) → Private Hospital (× 1)
P16	47-year-old married Janajati male, education Grade 3, occupation foreign employment	Startle response, breathing difficulty, dizziness, insomnia, headache, trembling of body, palpitations, high blood pressure, heaviness of head, bodily discomfort, burning sensation, excessive sweating, pulsating sensation in head, throbbing headache, blurred vision, fearfulness	- Financial obligations and family responsibilities - Spiritual cause	Neighbouring country (India ×3) → Private Clinic (× 1) → Traditional Healer (× 1) → Private Hospital (× 1)
P8	28-year-old married Brahmin female, education Grade 6, occupation homemaker	Headache, numbness of body, weakness, musculoskeletal pain, neck stiffness, forgetfulness, tingling, irritability, low self-esteem, social withdrawal, choking sensation, pain/swelling, palpitations, shivering, fainting, startle response, nausea/ vomiting, fatigue, social withdrawal, body pain, fearfulness	- Psychological distress after abortion - Father’s illness - Stress related to husband’s occupation in the police	Private Clinic (× 1) → Traditional Healer (× 1) → Primary Health Care Facilities (× 1) → Traditional Healer (× 1) → Primary Health Care Facilities (× 1) → Private Hospital (× 1) → Traditional Healer (× 1) → Primary Health Care Facilities (× 3)
P17	50-year-old married Brahmin female, education Grade 4, occupation farmer	Cold intolerance, body pain, reduced appetite, swollen hands and feet, insomnia, fatigue, fear, restlessness, loneliness, suffocation, crying, screaming, dissociative spells (sudden strangulation)	- Marital problems (domestic violence from husband) - Family conflict with sister-in-law and mother-in-law	Traditional Healers (× 3) → Primary Health Care Facilities (× 1) → Private Clinic (× 1) → Traditional Healer (× 1) → Primary Health Care Facilities (× 1) → Private Clinic (× 1) → Primary Health Care Facilities (× 1)
P3	19-year-old unmarried Brahmin female, education Grade 12, occupation student	Abdominal pain, fearfulness, shivering, fear of social setting, chest pain, thoughts of self-harm, anxious foreboding, excessive thoughts, social withdrawal, loss of interest, easily upset over trivial matters, irritability	- Conflict with mother and other relatives - Sexual harassment by a relative - Emotional distress in absence of father	Primary Health Care Facilities (× 1) → Traditional Healer (× 1) → Primary Health Care Facilities (× 1) → Private Hospital (× 1) → Primary Health Care Facilities (× 1) → Government Hospital (× 1)
P4	44-year-old married Dalit female, education Grade 8, occupation homemaker	Attempted self-harm, dissociative spells (trying to self-strangulate, bodily movements), getting overwhelmed, chest pain, suffocation, fearfulness, startle, palpitation, restlessness, not caring about others *“kasai prati maya nai nalagney”*, insomnia	- Bereavement (Brother-in-law’s illness and death) - Financial difficulties	Traditional Healers (× 11) → Private Hospital (× 1) → Primary Health Care Facilities (× 1) → Government Hospital (× 1)
P5	34-year-old married Dalit male, education bachelor’s degree, occupation gold shop/business	Insomnia, persistent fearfulness, low self-confidence, irritability, social isolation, body aches, fatigue, heaviness of body, difficulty concentrating, feeling unsteady, racing thoughts, pulsating sensation of head, fear that one would be killed, negative thoughts	- Homesickness after moving abroad - Family and community beliefs in spiritual causes and ghosts	Health Facility in Neighbouring Country (India) (× 1) → Private Hospital (× 1) → Traditional Healer (× 1) → Government Hospital (× 1) → Private Hospital (× 1)
P24	30-year-old married Chhetri female, education grade 10, occupation homemaker	Gastrointestinal, upset (diarrhea, vomiting), sticky sensation (throat) *“ghaati bijhairakhney”,* head numbness, racing thoughts, suffocation, limb tingling, body numbness, cold extremities, loss of appetite	- Persistent focus on gastritis related illness	Private Clinic (× 1) → Private Hospital (× 1) → Traditional Healers (× 2) → Private Hospital (× 1) → Neighbouring Country (× 1) → Government Hospital (× 1) → Traditional Healer (× 1) → Private Hospital (× 1)
P18	58-year-old married Chhetri female, education Grade 10, occupation business	Palpitation, headache, burning sensation in ear, anxiety *“maan attiney”*, strangulation, restlessness, increased blood pressure, decreased appetite, anhedonia, throbbing sensation in head, startle response, fearfulness	- Financial crisis and inability to repay loans due to lockdown	Primary Health Care Facilities (× 1)
P15	58-year-old married Chhetri female, education literate, occupation farmer	Social withdrawal, restlessness, insomnia, dissociation spells *(chopcha, lagu bhako jasto)*, excessive thoughts, crying spells, joint pain, fainting, low-confidence, hypervigilance, burning sensation, anxious foreboding, startle response	- Son’s accident and lack of support from husband - Husband’s accident - Problem in daughter’s marriage	Government Hospital (× 2) → Primary Health Care Facility (× 1)
P10	18-year-old unmarried Dalit male, education Grade 12, occupation student	Fearfulness, burning sensation in head, overwhelmed, palpitation, anhedonia, negative thoughts, fear about losing control	- Fear of insanity triggered by childhood snake sightings and related cultural images (e.g., Nagin poster)	Primary Health Care Facility (× 1) → Traditional Healer (× 1) → Private Clinic (× 1) → Primary Health Care Facility (× 1)
P2	30-year-old married Tharu male, education bachelor third year, occupation social mobilizer	Loss of appetite, fatigue, GI upset (indigestion, vomiting, nausea), sexual dysfunction, tension and worry, headache/ head bursting sensation, numbness of lower limbs (legs), shortness of breath, generalized weakness, shivering, fear of impending doom, burning sensation in body, restlessness	- Bereavement (Father’s death, Sister’s suicide) - Mother’s illness - Financial problems	Private Clinic (× 1) → Traditional Healer (× 1) → Neighbouring Country (× 1) → Government Hospital (× 1) → Neighbouring Country (× 1) → Private Hospital (× 2) → Health Camp (× 1)
P21	51-year-old widow Brahmin female, education Grade 7, occupation housewife	Persistent crying, dissociation spells (seeing shadows, illusion of someone calling at night, strange dreams), palpitations, loss of appetite, dryness throat, difficulty breathing, insomnia, fearfulness, thought of self-harm, tingling in hands/feet, feverish sensation, forgetfulness, heaviness of head, bursting sensation of head, pain and numbness, blurry vision, dizziness, fatigue, GI upset (stomachache/ heartburn)	- Bereavement (Husband’s death) - Conflict and misbehavior from daughter-in-law	Neighbouring Country (× 1) → Traditional Healer (× 1) → Government Hospital (× 1) → Private Hospital (× 2) → Health Camp (× 1)
P14	31-year-old married Dalit female, education Grade 5, occupation homemaker	Tingling sensation (legs), palpitation, dizziness, fear of impending doom, fearfulness, headaches, startle response, dryness throat, repetitive thoughts, getting anxious after watching social media, abdomen bloating, insomnia, shivering	- Alcoholic and unemployed husband, not supporting family financially or emotionally - Family disputes, neglect and shouting from mother-in-law	Neighbouring Country (× 1) → Traditional Healer (× 1) → Private Hospital (× 1) → Primary Health Care Facility (× 2) → Private Hospital (× 1) → Government Hospital (× 1)
P13	49-year-old married Tharu female, education literate (Praudh Shikshya), occupation farmer	Palpitation, headache, excessive worry, ocular pain, limb numbness of limbs/nerves, burning sensation over body	- Past abduction by Maoists	Neighbouring Country (× 1) → Traditional Healer (× 1) → Government Hospital (× 1) → Primary Health Care Facility (× 1)
P12	47-year-old married Tharu female, education Grade 12, occupation teacher	Fearfulness, startle response, shivering head, fearfulness, palpitations, insomnia, loss of appetite, headache, feeling that mind is unable to think or decide, fear of impending doom intrusive negative thoughts, generalized bodily pain	- Bereavement (Death of father-in-law and brother-in-law) - Financial fraud involving another brother-in-law	Health Camp (× 1)
P1	33-year-old married Dalit female, education Grade 6, occupation office assistant	Headache, body pain, GI upset, feeling that mind is unable to think or decide, negative thoughts, hopelessness, attempt at self-harm, fearfulness, shouting/ screaming, ocular pain, forgetfulness, difficulty breathing, palpitation, insomnia	- Financial problems - Bereavement (loss of husband) - Severe isolation during lockdown and lack of healthcare access - Pressure from family to remarry	Traditional Healers (× 2) → Government Hospital (× 1) → Traditional Healer (× 1) → Private Clinic (× 2) → Government Hospital (× 1) → Health Camp (× 1)
P11	22-year-old married Chhetri female, education grade 12 occupation beauty parlour	Fluctuating mood, attempt at self-harm, crying, poor concentration, forgetfulness, tinnitus, unsteadiness of body, inflicting pain and self-harm to get rid of sadness, neck stiffness, restlessness, physical discomfort, laziness, dizziness	- Husband’s extramarital affair - Marital conflict - Husband preventing work outside home	Primary Health Care Facility (× 2) → Government Hospital (× 1)
P20	30-year-old married Dalit female, education Grade 7, occupation farmer	Dissociation spells (ekkasi chope jasto huney; lagu bhako jasto), weeping, insomnia, feeling of emptiness, hopelessness, loss of interest, crying, restlessness, tingling and numbness in hands and legs, heaviness of body, fearfulness, difficulty breathing, numb nerves, loud shouting	- Mother went missing abroad - Bereavement (Death of father) - Anxiety related to health - Financial crisis - Marital fights due to money issues - Friend’s financial fraud	Traditional Healer (× 1) → Government Hospital (× 2) → Private Clinic (× 1) → Primary Health Care Facility (× 1)

### Symptoms experienced

Participants reported experiencing a combination of emotional, cognitive, and somatic symptoms. Initially, they experienced fearfulness, anxiety, restlessness, irritability, and heightened startle responses. Low self-confidence, social withdrawal, persistent sadness, and feelings of hopelessness were common. For example, a 60-year-old retired teacher shared his anxiety, worry, fear of crowded places, and feeling of overwhelming responsibility. Despite these challenges, he only discussed concerns about palpitation and tension with his family.

*I used to constantly feel scared and unhappy. Minor issues triggered self-doubt and deepened my sadness. Spending nights alone in a small hut heightened my loneliness and anxiety. I struggled to interact with others, had difficulty remembering things, and experienced disrupted sleep. I felt adrift and disconnected, lacking energy for daily activities and finding no pleasure in anything. Social gatherings filled me with fear and anxiety, and I questioned my value and purpose in everyday tasks. It was a challenging period, but I’m grateful to have overcome it.* ***P9***

Somatic symptoms such as headaches, gastrointestinal issues, and fatigue were also often mentioned as secondary effects of psychological stress. Some participants described specific symptoms, while others experienced a range of physical symptoms affecting different body systems, such as fainting, trembling, heaviness in the body, and difficulty breathing.

A 30-year-old married Tharu male facing financial difficulties, bereavement, and ongoing caregiving responsibilities, reported significant physical and psychological distress. His symptoms included generalized weakness, gastritis, sexual dysfunction, and a persistent fear of imminent death.

*Initially, I experienced gastritis followed by a sensation of coldness in my left leg that gradually spread upwards. My nerves weakened, rendering my body unable to function properly. Subsequently, I began to experience intense headaches, chest pain, body tremors, and difficulty breathing.* ***P2***

Similarly, a 47-year-old male migrant worker shared a similar experience, feeling pressurized to work despite being ill and concerned about job and financial security. He described experiencing sudden bouts of dizziness, fear, shortness of breath, and a feeling of impending death.

*While I was overseas, I encountered a strange problem. Whenever I was in a vehicle or trying to sleep, I would feel like the vehicle was moving on its own or reversing. This sensation would startle me awake, but then I would drift back to sleep. There was a moment when I felt a shock, as if the vehicle was in motion, and it frightened me. After some time, I would eventually fall back asleep. I would wake up abruptly, struggling to breathe. My chest felt constricted, making it difficult to take in air. I was afraid that I might not survive, recalling a Bengali friend who had died in a similar manner in my room. I experienced intense dizziness, feeling like the room was spinning, and I called out to my friends for help. They didn’t believe me, assuming it was due to alcohol. As I battled the dizziness, I couldn’t shake off the fear of meeting the same fate as my friend. I decided to take a cold shower to snap out of it. The cold water jolted my system.* ***P16***

Less common symptoms included dissociative episodes, where participants described brief periods of feeling detached, experiencing amnesia for certain events, or perceiving distortions such as shadows or a sensation of presence. While not experienced by everyone, those who did experience dissociation found it unsettling and sometimes confusing, with some noting a temporary loss of control over their body or surroundings. A smaller group of individuals associated these episodes with intense fear or panic, and a few linked dissociative experiences to instances of self-harm or unusual physical movements.

*I used to feel scared when I encountered ghosts, even though I was not scared when I went to the forest with my friends. People around me thought I was crazy. I had to confront ghosts not just at night but also during the day, and over time, this fear grew. I believe that fear is my illness. Right now, I am able to laugh and talk to you without any fear but when this illness resurfaces, the fear will return.* ***P19***

### Perceived cause of illness

Participants reported a variety of reasons for their distress or anxiety, often stemming from multiple life stressors that overlapped. Financial difficulties such as debts, the inability to repay loans, and managing household responsibilities, especially after the loss of a primary earner or during lockdowns, were frequently mentioned. Bereavement was also a common factor, with participants citing the death of father, husbands, in-laws, and siblings, sometimes compounded by other ongoing stressors. Trauma-related events such as abortion, sexual harassment, and childhood fears were also mentioned in some cases. Participants often connected multiple causes, indicating that distress was perceived as a result of intertwined financial, relational, health, and cultural factors rather than a single identifiable source.

A 47-year-old married woman shared the losses her family had faced since her husband disappeared after being arrested by the Nepal Army during the Maoist conflict. She expressed ongoing fear from past army harassment and current household stressors that were contributing to her emotional distress.

*I have lost my father-in-law and husband, and tragically, my youngest brother-in-law has passed away. My middle brother-in-law has also caused a lot of trouble. It’s hard to describe how challenging our situation has been. He took out multiple loans using our family property as collateral, and when he couldn’t repay the interest, the bank threatened to auction off our property. It was a nightmare for me, and I couldn’t understand why this was happening to us. My mind was not functioning properly and I couldn’t comprehend why we were going through this.* ***P12***

Several participants believed that the problems they experienced were caused by spiritual or supernatural forces, such as possession by ghosts. One female participant described how the spirit of her husband’s first wife was causing trouble for her.

*My husband’s first wife died by hanging herself, and her spirit keeps appearing, trying to take over my body. Sometimes, her spirit attacks me with convulsions, but I was not initially scared. I struggle witheating, moving, falling, and losing consciousness. When I start to fall, my husband accuses me of bringing illness from my family home, tells me to go back there, and even chases me and beats me.* ***P17***

### Health care practice

Participants in the study reported diverse help-seeking practices, reflecting both biomedical and traditional approaches. Initially, the majority (18 out of 24) sought care from biomedical providers, such as private clinics (n = 5), government hospitals (n = 2), private hospitals (n = 1), primary health care facilities (n = 4), healthcare facilities in a neighboring country (n = 5), and health camps (n = 1). In contrast, six participants (25%) initially consulted traditional healers.

Treatment pathways and practices varied based on symptom presentation. For those experiencing dissociative episodes (n = 7), the majority (n = 4) first sought help from a traditional healer, while others went to a government hospital (n = 2) or sought care in a neighboring country (n = 1). Participants with physical complaints (n = 6) typically started at a private clinic (n = 3), followed by facilities in a neighboring country (n = 2) and a primary health care facility (n = 1). Those with cognitive or emotional symptoms (n = 7) had mixed pathways, with some starting at private or government hospitals, while others visited primary health care facilities or traditional healers.

Subsequent consultations revealed dynamic and iterative patterns of care-seeking, with individuals frequently alternating between traditional, private, and public healthcare providers. While government hospitals and primary health care facilities were not typically the initial choice, they were often utilized for subsequent visits. Some participants integrated biomedical care from other countries with local services, while others alternated between primary health care facilities and traditional healers.

Table 2 summarizes the number of visits participants made to different types of providers. Each visit was counted individually; for example, if a participant visited the same provider twice, regardless of the sequence of visits, this was recorded as two separate visits. Traditional healers were the most frequently consulted providers, followed by private hospitals, and primary healthcare facilities. Visits to neighboring countries and attendance at health camps were less common but still represented components of some care trajectories.

**Table 2:** Number of visits with different service providers.

Service providers	Number of visits/sessions (N)	%
Traditional healers	38	27.7
Government hospital	18	13.1
Primary health care facilities	22	16.1
Private hospital	27	19.7
Private clinic	16	11.7
Health camp	5	3.7
Neighboring country mostly in India	11	8.0

Participants attributing symptoms to bereavement (n = 9) or family conflict (n = 11) often used both systems, though biomedical care was somewhat more common. Similarly, those citing financial stress (n = 12) showed mixed patterns. In contrast, trauma and culturally rooted fears (n = 11) strongly favored pluralistic care, with ten participants consulting both healers and biomedical providers.

Socio-demographic factors showed limited influence on help-seeking practices. Both men and women predominantly sought biomedical care in their first consultation (8 out of 9 males; 10 out of 15 females). Healthcare facilities were also the preferred choice across age groups: all younger participants (<30 years, n = 5), most middle-aged participants (30–50 years, 10 out of 14), and the majority of older adults (>50 years, 4 out of 5) initially consulted biomedical providers, although many later diversified their care.

Caste and educational status, however, showed clearer variation. Fewer Dalit and marginalized caste participants (4 out of 7) sought biomedical care initially compared to Brahman/Chhetri (10 out of 12), Janajati (1 out of 2), and all the Tharu participants (3 out of 3). Education strongly shaped pathways: all participants with Grade 12 or higher education (9 out of 9) first consulted biomedical providers, compared to only 60% (9 out of 15) of those with Grade 10 or lower education. Occupational differences were also observed; 12 of 14 participants employed in professions such as teaching, business, or foreign employment first sought biomedical care, whereas only 6 out of 10 participants engaged in agriculture or household work did so.

The study participants reported a wide range of emotional, cognitive, and physical symptoms, with fear, anxiety, sadness, restlessness, and hopelessness being the most common. Cognitive difficulties included memory issues, social withdrawal, and a sense of purposelessness, while physical complaints such as headaches, disrupted sleep, gastrointestinal problems, fatigue, chest pain, and dizziness reflected the physical burden of stress. Some participants also described dissociative episodes, often associated with fear, panic, or self-harm. The distress was linked to various life stressors like financial difficulties, loss of loved ones, family conflicts, caregiving responsibilities, trauma, and culturally ingrained fears, with some participants attributing their symptoms to supernatural causes. Participants used a mix of healthcare practices, engaging both biomedical and traditional systems. Most participants initially sought biomedical care while some consulted traditional healers. Many participants switched between providers, with traditional healers being the most frequently visited overall. Care-seeking pathways varied based on symptoms and perceived causes, but involved a combination of both systems, especially for trauma and culturally rooted fears. Socio-demographic factors such as gender and age had minimal impact, while education and caste significantly influenced initial care-seeking choices. Participants with higher education levels and from higher-castes tended to seek biomedical care first.

The study findings highlighted that depression and anxiety in Nepal are often characterized by a combination of emotional, cognitive, and physical symptoms influenced by cultural factors. Participants frequently reported feelings of fear, restlessness, excessive thinking, fatigue, and physical symptoms such as palpitations, chest tightness, and gastrointestinal upsets. The physical symptoms reported in our study are consistent with previous research indicating that in Nepal, expressing distress through physical symptoms is more socially acceptable than discussing emotions directly, due to the stigma associated with mental health issues [31–33]. Fear and negative thoughts were identified as key cognitive symptoms exacerbating anxiety and depression, reflecting culturally significant ways of expressing distress [34].

Participants’ explanatory models were influenced by culturally ingrained fears and supernatural beliefs, leading many to attribute their symptoms to spirits or other malevolent forces. This often led them to seek initial consultations with traditional healers, a pattern consistent with previous studies [24,35]. Consequently, traditional healers were the preferred first point of care for many participants, especially those who believed their symptoms were linked to supernatural forces, trauma, or culturally defined illnesses. Even after accessing biomedical services, many individuals continued to consult traditional healers, a pattern commonly observed among people seeking mental healthcare [24,36]. This underscores the enduring cultural trust and significance of traditional healing practices in Nepali communities, where spiritual explanations for mental distress remain highly influential [37,38].

Pluralistic care-seeking, involving both traditional healers and biomedical providers, was common, especially for trauma and culturally attributed causes. Participants often sought care from both traditional healers and biomedical providers, either sequentially or simultaneously, reflecting a practical and culturally informed approach to managing distress. Traditional healers were typically consulted first for issues perceived as spiritual or attributed to supernatural forces, while biomedical services were sought for physical symptoms or when traditional treatments did not yield results. This is consistent with previous studies in Nepal that have shown mental health care pathways often incorporate both cultural and biomedical elements [39–41]. Similar pluralistic practices have been observed in other South and East Asian contexts, where culturally relevant beliefs influence help-seeking behaviors and treatment adherence [42,43]. Importantly, this trend aligns with broader research on consumer behavior in healthcare, which suggests that individuals actively evaluate and seek value from multiple care options rather than passively receiving services [44].

Education and caste play a significant role in shaping individuals’ initial treatment choices. Education strongly influences help-seeking patterns; individuals with higher levels of education are more likely to turn to medical professionals or healthcare facilities compared to those with lower levels of education. Furthermore, individuals with secondary education or higher did not attribute their mental health issues to supernatural causes, instead linking them to factors such as economic hardships, the loss of a loved one, suicide in family, or other traumatic events. This aligns with research in South Asia indicating that education improves mental health literacy, awareness of available services, and trust in biomedical care [45]. Similarly, caste and social status impact access to healthcare services and perceptions of different treatment options, which may explain why individuals from marginalized groups often seek traditional healers as their first choice [46].

### Implications

The findings of this study have significant implications for early detection and improvement of help-seeking practices among individuals with depression and anxiety in Nepal. First the study revealed that people in Nepal often seek care from both traditional healers *(such as dhami, jhakri,* or *lama)* and biomedical providers, reflecting a diverse and culturally-informed care-seeking approach influenced by cultural beliefs and accessibility issues. Closer collaboration between local health facilities and traditional healers could enhance early detection and mental health outcomes. Establishing reliable referral pathways and providing basic training to traditional healers to recognize symptoms of depression and anxiety could further support early detection and treatment. Second, education, and caste/ethnicity were found to significantly influence individuals’ initial help-seeking decisions. Dalit and marginalized groups experience stigma, discrimination, and limited access to mental health information [47], underscoring the needs for community-based mental health literacy initiatives. These programs should be delivered through accessible and trusted community actors such as mothers’ groups, FCHVs, and traditional healers [39]. Third, the results indicated that bereavement, particularly the loss of loved ones, was a major trigger for depression and anxiety in Nepal’s close-knit family and community-oriented culture. Implementing trauma-informed interventions within communities, such as peer support groups, culturally sensitive grief counseling, and integrating psychosocial support with traditional mourning practices, could help individuals cope with their grief effectively [48]. Finally, family conflicts and financial difficulties were common contributors to depression and anxiety, with women disproportionately affected by domestic violence, intergenerational disputes, and economic hardships. Addressing these challenges requires comprehensive community-based interventions that combine psychological, social and economic/livelihood support. Programs that prioritize conflict resolution, family counseling, and women’s support groups can offer safe environments to address abuse and promote empowerment.

### Strengths and limitations

The study has several strengths. First, to the best of our knowledge, this is the first study in Nepal to explore the relationship between symptom presentation, perceived causes of illness, and help-seeking practices. Second, participants were selected from three different regions of Nepal (eastern, central, and western), representing diverse socio-economic backgrounds. Third, the study included individuals with depression or anxiety, recruited through various healthcare providers. Lastly, the use of the McGill Illness Narrative Interview (MINI), allowed for detailed narratives about symptom experiences, illness stories, and help-seeking practices.

The study also has a number of limitations. First, it was carried out with a purposively selected sample size of 24 participants undergoing treatment for depression or anxiety disorders, which may limit the generalizability of the results. Second, participants were still in treatment during the interviews, potentially not providing a complete picture of the treatment process. Lastly, the study was conducted in areas with specialized mental health services which may affect the generalizability of the results to regions lacking these services.

## Conclusion

The findings revealed that depression and anxiety in Nepal manifest as a mix of emotional, cognitive, and somatic symptoms, influenced by traumatic events, cultural fears and supernatural beliefs. Common symptoms include anxiety, sadness, and hopelessness, as well as somatic complaints and occasional dissociative episodes, highlighting the severity of individuals’ distress. Many individuals seek help from both traditional healers and biomedical providers for treatment, particularly for trauma and culturally-specific issues, with education and caste shaping their treatment-seeking choices. The study underscores the need for culturally sensitive mental health services that promote collaboration between biomedical providers and traditional healers to enhance access and acceptance of care. Tailored community-based mental health literacy programs are essential for different caste and education groups to combat stigma, promote early detection, and encourage timely help-seeking.

## Supporting information

S1 Table Coding Framework. This table outlines the coding framework used in the qualitative analysis. Codes were developed multiple reviews of de-identified interview transcripts and refined through team discussions. The final codes were grouped into broader thematic domains: somatic, emotional, cognitive, behavioural, and cultural symptoms. Examples of codes definitions and verbatim quotations are provided to illustrate theme development from the data.
(DOCX)

## References

[1] WHO. Depression and other common mental disorders: global health estimates. Geneva: World Health Organization. 2017.

[2] COVID-19 Mental Disorders Collaborators. Global prevalence and burden of depressive and anxiety disorders in 204 countries and territories in 2020 due to the COVID-19 pandemic. Lancet. 2021;398(10312):1700–12. 34634250 10.1016/S0140-6736(21)02143-7 PMC8500697

[3] Remes O, Mendes JF, Templeton P. Biological, psychological, and social determinants of depression: a review of recent literature. Brain sciences. 2021;11(12). 10.3390/brainsci11121633 PMC8699555 34942936

[4] Peerenboom L, Collard RM, Naarding P, Comijs HC. The association between depression and emotional and social loneliness in older persons and the influence of social support, cognitive functioning and personality: A cross-sectional study. J Affect Disord. 2015;182:26–31. doi: 10.1016/j.jad.2015.04.033 25965692

[5] Peyrot WJ, Lee SH, Milaneschi Y, Abdellaoui A, Byrne EM, Esko T, et al. The association between lower educational attainment and depression owing to shared genetic effects? Results in ~25,000 subjects. Mol Psychiatry. 2015;20(6):735–43. doi: 10.1038/mp.2015.50 25917368 PMC4610719

[6] Brandt L, Liu S, Heim C, Heinz A. The effects of social isolation stress and discrimination on mental health. Transl Psychiatry. 2022;12(1):398. doi: 10.1038/s41398-022-02178-4 36130935 PMC9490697

[7] Ormel J, Cuijpers P, Jorm A, Schoevers RA. What is needed to eradicate the depression epidemic, and why. Mental Health & Prevention. 2020;17:200177. doi: 10.1016/j.mhp.2019.200177

[8] Buus N, Petersen A, McPherson S, Meadows G, Brand G, Ong B. The relatives of people with depression: A systematic review and methodological critique of qualitative studies. Fam Process. 2024;63(3):1469–83. doi: 10.1111/famp.12927 37604511

[9] Keynejad R, Spagnolo J, Thornicroft G. WHO mental health gap action programme (mhGAP) intervention guide: updated systematic review on evidence and impact. Evid Based Mental Health. 2021;24(3):124–30. doi: 10.1136/ebmental-2021-300254 PMC8311089 33903119

[10] Singla DR, Kohrt BA, Murray LK, Anand A, Chorpita BF, Patel V. Psychological Treatments for the World: Lessons from Low- and Middle-Income Countries. Annual Review of Clinical Psychology. 2017;13:149–81. 10.1146/annurev-clinpsy-032816-045217 PMC5506549 28482687

[11] Mekonen T, Chan GCK, Connor JP, Hides L, Leung J. Estimating the global treatment rates for depression: A systematic review and meta-analysis. J Affect Disord. 2021;295:1234–42. doi: 10.1016/j.jad.2021.09.038 34665135

[12] Thornicroft G, Chatterji S, Evans-Lacko S, Gruber M, Sampson N, Aguilar-Gaxiola S, et al. Undertreatment of people with major depressive disorder in 21 countries. Br J Psychiatry. 2017;210(2):119–24. doi: 10.1192/bjp.bp.116.188078 27908899 PMC5288082

[13] van den Broek M, Gandhi Y, Sureshkumar DS, Prina M, Bhatia U, Patel V, et al. Interventions to increase help-seeking for mental health care in low- and middle-income countries: A systematic review. PLOS Glob Public Health. 2023;3(9):e0002302. doi: 10.1371/journal.pgph.0002302 37703225 PMC10499262

[14] Zhang R, Peng X, Song X, Long J, Wang C, Zhang C, et al. The prevalence and risk of developing major depression among individuals with subthreshold depression in the general population. Psychol Med. 2023;53(8):3611–20. doi: 10.1017/S0033291722000241 35156595 PMC10277767

[15] Fekadu A, Demissie M, Birhane R, Medhin G, Bitew T, Hailemariam M, et al. Under detection of depression in primary care settings in low and middle-income countries: a systematic review and meta-analysis. Syst Rev. 2022;11(1):21. doi: 10.1186/s13643-022-01893-9 35123556 PMC8818168

[16] Kleinman A. Culture and depression. N Engl J Med. 2004;351(10):951–3. doi: 10.1056/NEJMp048078 15342799

[17] Pudasaini K, Khadgi R, Badu J, Gautam K, Kohrt BA, Luitel NP. Somatic symptoms still matter: A qualitative study of lived experience of depression in Nepal. Transcultural Psychiatry.

[18] Kohrt BA, Hruschka DJ. Nepali concepts of psychological trauma: the role of idioms of distress, ethnopsychology and ethnophysiology in alleviating suffering and preventing stigma. Cult Med Psychiatry. 2010;34(2):322–52. doi: 10.1007/s11013-010-9170-2 20309724 PMC3819627

[19] Kirmayer LJ, Young A. Culture and somatization: clinical, epidemiological, and ethnographic perspectives. Psychosom Med. 1998;60(4):420–30. doi: 10.1097/00006842-199807000-00006 9710287

[20] Ryder AG, Yang J, Zhu X, Yao S, Yi J, Heine SJ. The cultural shaping of depression: somatic symptoms in China, psychological symptoms in North America?. Journal of Abnormal Psychology. 2008;117(2):300–13. 18489206 10.1037/0021-843X.117.2.300

[21] Sagar-Ouriaghli I, Godfrey E, Bridge L, Meade L, Brown JSL. Improving Mental Health Service Utilization Among Men: A Systematic Review and Synthesis of Behavior Change Techniques Within Interventions Targeting Help-Seeking. Am J Mens Health. 2019;13(3):1557988319857009. doi: 10.1177/1557988319857009 31184251 PMC6560805

[22] Chang Q, Yip PSF, Chen Y-Y. Gender inequality and suicide gender ratios in the world. J Affect Disord. 2019;243:297–304. doi: 10.1016/j.jad.2018.09.032 30261445

[23] WHO. Global Health Observatory (GHO) data. World Health Organization. 2017. https://www.who.int/gho/mental_health/suicide_rates_male_female/en/

[24] Gupta AK, Joshi S, Kafle B, Thapa R, Chapagai M, Nepal S, et al. Pathways to mental health care in Nepal: a 14-center nationwide study. Int J Ment Health Syst. 2021;15(1):85. doi: 10.1186/s13033-021-00509-4 34930398 PMC8685796

[25] Luitel NP, Lamichhane B, Pokhrel P, Upadhyay R, Taylor Salisbury T, Akerke M, et al. Prevalence of depression and associated symptoms among patients attending primary healthcare facilities: a cross-sectional study in Nepal. BMC Psychiatry. 2024;24(1):356. doi: 10.1186/s12888-024-05794-0 38745133 PMC11092057

[26] Pradhan U, Koirala N, Shrestha M, Parajuli SB. Pathways to mental health care services among patients in hospitals of Morang district, Nepal. J Karnali Academy of Health Sciences. 2022;5(2).

[27] Hunter D, McCallum J, Howes D. Defining exploratory-descriptive qualitative (EDQ) research and considering its application to healthcare. J Nursing and Health Care. 2019;4(1).

[28] Lincoln YS, Guba EG. Naturalistic inquiry. sage. 1985.

[29] Groleau D, Young A, Kirmayer LJ. The McGill Illness Narrative Interview (MINI): an interview schedule to elicit meanings and modes of reasoning related to illness experience. Transcult Psychiatry. 2006;43(4):671–91. doi: 10.1177/1363461506070796 17166953

[30] Craig SR, Chase L, Lama TN. Taking the MINI to Mustang, Nepal: methodological and epistemological translations of an illness narrative interview tool. Anthropol Med. 2010;17(1):1–26. doi: 10.1080/13648471003602566 20419514

[31] Hoge EA, Tamrakar SM, Christian KM, Mahara N, Nepal MK, Pollack MH, et al. Cross-cultural differences in somatic presentation in patients with generalized anxiety disorder. J Nerv Ment Dis. 2006;194(12):962–6. doi: 10.1097/01.nmd.0000243813.59385.75 17164637

[32] Kohrt BA. “Somatization” and “Comorbidity”: A Study of Jhum-Jhum and Depression in Rural Nepal. Ethos. 2005;33(1):125–47. doi: 10.1525/eth.2005.33.1.125

[33] Belbase M, Adhikari J, Khan TA, Jalan RK. Depression with Somatic Symptoms in Patients Attending Psychiatry OPD of Nepalgunj Medical College. J Nepalgunj Med College. 2016;12(2):17–9. doi: 10.3126/jngmc.v12i2.14470

[34] Mendenhall E, Rinehart R, Musyimi C, Bosire E, Ndetei D, Mutiso V. An ethnopsychology of idioms of distress in urban Kenya. Transcult Psychiatry. 2019;56(4):620–42. doi: 10.1177/1363461518824431 30672722

[35] Kate N, Grover S, Kulhara P, Nehra R. Supernatural beliefs, aetiological models and help seeking behaviour in patients with schizophrenia. Ind Psychiatry J. 2012;21(1):49–54. doi: 10.4103/0972-6748.110951 23766578 PMC3678179

[36] Nortje G, Oladeji B, Gureje O, Seedat S. Effectiveness of traditional healers in treating mental disorders: a systematic review. Lancet Psychiatry. 2016;3(2):154–70. doi: 10.1016/S2215-0366(15)00515-5 26851329

[37] Luitel NP, Lamichhane B, Koirala P, Sainju P, Ghimire A, Gautam K, et al. Treatment of depression by traditional faith healers in Nepal: A qualitative study. SSM Ment Health. 2025;7:100425. doi: 10.1016/j.ssmmh.2025.100425 40519523 PMC12163705

[38] Pham TV, Koirala R, Wainberg ML, Kohrt BA. Reassessing the mental health treatment gap: what happens if we include the impact of traditional healing on mental illness?. Community Mental Health Journal. 2021;57(4):777–91. 32894398 10.1007/s10597-020-00705-5 PMC7936992

[39] Luitel NP, Lamichhane B, Sah K, Basnet B, Sainju P, Gautam K, et al. Facilitators in treatment pathways for depression or anxiety among adults in Nepal: a qualitative study. BMC Public Health. 2025;25(1):2033. doi: 10.1186/s12889-025-23225-x 40457314 PMC12128395

[40] Pham TV, Koirala R, Kohrt BA. Traditional and biomedical care pathways for mental well-being in rural Nepal. Int J Ment Health Syst. 2021;15(1):4. doi: 10.1186/s13033-020-00433-z 33413540 PMC7792081

[41] MacDonald K, Fainman-Adelman N, Anderson KK, Iyer SN. Pathways to mental health services for young people: a systematic review. Soc Psychiatry Psychiatr Epidemiol. 2018;53(10):1005–38. doi: 10.1007/s00127-018-1578-y 30136192 PMC6182505

[42] Febriyanti RM, Saefullah K, Susanti RD, Lestari K. Knowledge, attitude, and utilization of traditional medicine within the plural medical system in West Java, Indonesia. BMC Complementary Medicine and Therapies. 2024;24(1):64. 38287364 10.1186/s12906-024-04368-7 PMC10826289

[43] Williams SA, Baldeh M, Bah AJ, Dennis F, Robinson DR, Adeniyi YC. Pathways to mental health services across local health systems in sub-Saharan Africa: Findings from a systematic review. PLoS One. 2025;20(6):e0324064. doi: 10.1371/journal.pone.0324064 40526705 PMC12173185

[44] Gordon D, Ford A, Triedman N, Hart K, Perlis R. Health care consumer shopping behaviors and sentiment: qualitative study. J Particip Med. 2020;12(2):e13924. doi: 10.2196/13924 33064088 PMC7434061

[45] Subu MA, Holmes D, Arumugam A, Al-Yateem N, Maria Dias J, Rahman SA, et al. Traditional, religious, and cultural perspectives on mental illness: a qualitative study on causal beliefs and treatment use. Int J Qual Stud Health Well-being. 2022;17(1):2123090. doi: 10.1080/17482631.2022.2123090 36097886 PMC9481114

[46] Thapa R, van Teijlingen E, Regmi PR, Heaslip V. Caste Exclusion and Health Discrimination in South Asia: A Systematic Review. Asia Pac J Public Health. 2021;33(8):828–38. doi: 10.1177/10105395211014648 34024157 PMC8592103

[47] French AN. Dalits and mental health: investigating perceptions, stigma and barriers to support in Kathmandu, Nepal. Journal of Global Health Reports. 2020;4. doi: 10.29392/001c.12136

[48] Hendrickson ZM, Kim J, Tol WA, Shrestha A, Kafle HM, Luitel NP, et al. Resilience Among Nepali Widows After the Death of a Spouse: “That Was My Past and Now I Have to See My Present”. Qual Health Res. 2018;28(3):466–78. doi: 10.1177/1049732317739265 29110564

