# Supplementary material for: Symptom presentation, perceived causes, and help-seeking practices among adults receiving depression or anxiety care in Nepal: A qualitative study
Source: PLoS One. 2026 May 8;21(5):e0347605. doi: 10.1371/journal.pone.0347605 (PMC13155605; doi:10.1371/journal.pone.0347605)
Supplement: S1 Table — This table outlines the coding framework used in the qualitative analysis. Codes were developed multiple reviews of de-identified interview transcripts and refined through team discussions. The final codes were grouped into broader thematic domains: somatic, emotional, cognitive, behavioural, and cultural symptoms. Examples of codes definitions and verbatim quotations are provided to illustrate theme development from the data. (DOCX) [file pone.0347605.s001.docx]

Supplementary table 1 coding framework

| Symptoms presented | Description of symptoms | Quotations |
| --- | --- | --- |
| Somatic symptoms | | |
| Palpitations | An unusually fast or pounding heartbeat, often described as the heart "wrenching" or "shaking." | I used to have heart palpitation (*mutu dhukdhuk huney*). I used to be terrified. |
| Difficulty in breathing | Feeling of gasping for air, shortness of breath. | Sometimes my breathing would stop... my heart used to beat fast (*mutu dhuk dhuk huney*), and I used to feel heavy weight on my chest. |
| Suffocation | “Choking" sensation in the throat | I used to feel like something was stuck in my neck. |
| Neck stiffness | Physical tension or pain in the neck and shoulder area, often linked to high-stress levels. | This also used to be stiff... the neck used to be stiff... I have started to feel that there aren’t any parts of my head. |
| Fatigue | Extreme tiredness, lack of energy, or "lethargy" that prevents physical activity. | When I hold small objects, my hands quickly become tired |
| Weakness | reduced physical strength or internal bodily frailty, often described as the “nerves” or “heart” becoming weak, resulting in difficulty walking, standing, or performing routine tasks despite no visible injury. Example; *Nasa Kamjori,* *mutu kamjori* | My nerves were too weak and my body was unable to work... I used to walk like a tortoise wearing a shawl. |
| Appetite loss | A significant reduction in the desire to eat, sometimes accompanied by nausea or vomiting. | I've lost my interest in eating; I just take meals so that I can stand. |
| Burning sensation | Heat or stinging pain in the head, eyes, or throughout the body, often compared to "chili." | My body used to burn... I used to feel like being burnt by chilies. |
| Numbness | Physical sensations where limbs or parts of the body feel "dead," "immobile," or "fall asleep" (*nidaune*) without a biomedical explanation. | My legs here became as if they couldn't move even when I lifted them, I could not lift. |
| Tingling (*Jhamjham*) | Sensations of "electric current," pins and needles, or a "twinkling" feeling in the nerves or limbs, often described using the local idiom *siring siring*. | My hands and legs used to have tingling sensation (*Siring, siring huney*)... I felt like someone was holding me. |
| Headache | Persistent throbbing, "piercing" pain, or a feeling that the head might "blast." | My head started paining very badly, my head used to feel like it would blast. |
| Increased blood pressure | Stress-induced spikes in blood pressure measurements during periods of high anxiety. | At the health post, they took my vitals and said that my blood pressure was quite high, maybe due to stress. |
| Dizziness | A state of unsteadiness or light-headedness, often described as the sensation that the room is "rotating" or that one is about to "fall down," typically occurring during panic attacks or peak emotional worry. | I felt a heavy dizziness... felt like the room was rotating. |
| Blurred vision | Visual disturbances or a loss of clarity in sight, sometimes accompanied by severe headaches or a sensation of the world becoming "dark" or "frozen" during intense distress. | I am experiencing eye problems symptoms like blurry vision, headaches, and dizziness. |
| Cold intolerance | Feeling physically chilled or cold on the inside regardless of external temperature. | Now I’m feeling cold as ice, cold on the inside. |
| Excessive sweating | Profuse sweating often occurring alongside "cold blood" or heart palpitations. | I feel very hot while walking... Sweat breaks out amply and I go to check my pulse but they say 'you are fine’. |
| Abdominal pain | Sharp or nagging pain in the stomach region, often described as "*poking*" or associated with "gas." | There used to be a sharp pain in my inner stomach... the stomach used to feel a sharp pain from earlier. |
| Chest pain | Sharp, heavy, or burning sensation in the thoracic region, often mistaken for heart problems. | I used to feel heavy weight on my chest... my heart used to burn like a burning fire. |
| Gastrointestinal (GI) upset | Digestive disturbances including diarrhoea, vomiting, nausea, and indigestion linked to stress. | I got sick so badly that I didn’t eat anything for a week, even if I eat I would vomit all of it, indigestion. |
| Choking sensation | A feeling of something "stuck" in the throat or "irritating" the neck area. | I feel like I have choked on something... it feels like something is stuck over there. |
| Cold extremities | A physical sensation of the hands or legs becoming "icy" or "frozen." | My left leg started getting cold... slowly the feeling of cold moved upwards. |
| Ocular pain | Severe pain in or behind the eyes, often described as eyes "popping out." | My eyes used to feel extreme pain as if they would pop out... the pain in my head destroyed my eyes. |
| Tinnitus | Ringing, "bell" sounds, or burning sensations inside the ears. | It felt like a sound was coming through my ear... as if a bell was ringing. |
| Feverish sensation | A subjective feeling of rising body temperature or "fire" without a clinical infection. | I felt a rising temperature/fever... I used to say 'Mom... on the top of my head... rice may be cooked too’. |
| Musculoskeletal pain | Aches in the back, knees, elbows, or soles of the feet without injury. | I have extreme pain in my legs... in my knees, elbows, and the sole of my feet. |
| Heaviness of body | A sensation of physical lethargy or being "weighed down." | My body feels heavy to me, and at times I feel light. |
| Dizziness | A sense of being off-balance or feeling like the room is rotating. | I felt a heavy dizziness... felt like the room was rotating... I felt as if my legs were not walking in balance. |
| Emotional symptoms | | |
| Fearfulness | Pervasive anxiety and a constant sense of dread or terror regarding death or safety. | I am afraid in my heart-mind... I’m afraid that if I say these things, it will be known in my house. |
| Sadness | A profound sense of dejection, daily crying | The mind never felt happy... the mind keeps on getting sad about that problem. |
| Hopelessness | The feeling that life has no worth. | After that happened, I used to feel like there was nothing left but death... I felt like I wouldn’t live now, it will be too much, and everything will be finished. |
| Startle response (*Jhaskine*) | Being easily frightened by sudden noises, movements, or phone ringtones. | I get startled even by the ringtone of the phone. |
| Feeling lonely | A sense of social isolation or being unsupported by family and society. | Staying there [guarding the house alone] for a long time, I started to feel lonely. |
| Irritability | Sudden outbursts of anger or low tolerance for others' speech or behavior. | If they don’t obey me, I get very angry... I get irritated while talking with anyone. |
| Panic attacks | Sudden episodes of intense fear that trigger severe physical reactions (heart pounding/shaking). | There was a panic attack, I got that suddenly due to some reason. |
| Anxious foreboding (*Maan Attine*) | A state of persistent worry where the mind feels "nervous" or unsettled. | My heart feels fear or scared... my heart is not working properly... my mind becomes anxious. |
| Overwhelmed | Feeling unable to manage the intensity of financial or family pressure. | The situation was torturous to me. My brain stopped working properly. |
| Not caring about others | A loss of emotional attachment even toward close family members (*Kasai prati maya nai nalagne*). | I don’t feel care towards anyone... not even towards my husband. My mind doesn’t go towards anyone’s love. |
| Fear of impending doom / death | Intense terror that one is about to die or be killed. | I was feeling that I will not live any longer, I will die... someone might come and murder me. |
| Fear of losing control | Worry about acting "abnormally" or "becoming mad" in front of others. | I feared of being a mad person myself... imaginations started again about what would happen if I became mad. |
| Feeling of emptiness | A profound sense of loss or internal void. | My heart was feeling empty and uneasy... I feel like I am dead from the inside. |
| Hypervigilance | Being extremely sensitive to sounds or social environments. | I get startled even by the ringtone of the phone... I felt irritated just by hearing people speak. |
| Cognitive symptoms | | |
| Excessive thoughts | Racing, uncontrollable, and usually negative thoughts that occur most intensely at night. | I think the reason my head felt heavy was because I was overthinking. |
| Forgetfulness | Difficulty remembering names, misplacing items, or losing one's sense of direction. | I forget where I kept my glasses... I have been forgetting things faster lately. |
| Low self-esteem | Feelings of worthlessness or being "backward" and unable to make decisions. | My self-confidence is weak... I feel like I might not be able to do anything. |
| Difficulty concentrating | Inability to focus on tasks, work, or follow a conversation properly. | I can concentrate, but... it takes time for me to understand something... a second or two to process. |
| Vacant stares (*Tolaune*) | Periods of being absent-minded or staring blankly while lost in thought. | Some people feel absent-minded/constantly stare at something. They think of certain things and wander about them. |
| Repetitive thoughts | Uncontrollable cycles of negative thinking, often about the past or debt. | I have one thought playing in my mind over and over again... more from a negative perspective. |
| Unable to think or decide | Mental paralysis where one cannot make choices or understand simple instructions. | I reached a condition where I couldn’t even make a decision for myself... it takes time for me to process things. |
| Confusion | Losing one's sense of place or direction during periods of high stress. | I forget the way where I was supposed to go. I feel like where I'm now, where I'm going. |
| Anxiety after social media | Distress triggered by watching news or videos on a mobile phone. | Watching content on my phone... added up the stress with posts of some people dead. |
| Behavioural symptoms | | |
| Suicide attempt | Clear thoughts or physical actions (such as hanging, poison, or drowning) taken with the specific intent to end one's life because there is "no point in living". | I felt like should I have poison, should I die? At that time, I don’t feel love towards anyone... I feel like I am dead from the inside. |
| Insomnia | Persistent inability to fall asleep or stay asleep due to racing thoughts. | For eighteen days exactly, my eyes didn't join or I could not sleep. |
| Social isolation | Avoiding gatherings, refusing to speak to friends, and isolating oneself in dark rooms. | I stayed alone in school... didn't talk much... wanted to stay in solitude. |
| Crying | Outward vocalization of distress, often uncontrolled and occurring in public or alone. | I used to want to cry out loud. I wept loudly in the middle of the road. |
| Attempted self-harm | Physical actions taken to cause injury or end life (e.g., rope, poison, wrist-cutting). | I placed rope around my neck... I also tried taking 10-12 medications [at once]. |
| Fluctuating mood | Rapid shifts between extreme happiness and profound sadness. | I become very happy at small moments... and also get very sad at small things. |
| Laziness | A lack of will or energy to perform even basic daily chores. | My body wouldn’t get up... I feel very lazy at times... I felt like I was feeling laziness. |
| Cultural symptoms/presentation | | |
| Dissociative spells | Altered states of consciousness interpreted as fighting shadows or being "caught" by spirits, Vivid nightmares interpreted as visitations from the deceased or "witches." | The shadow would come and I would fight with it... fighting with shadows, fighting ghosts. |
| Loss of consciousness | Sudden blackouts attributed to supernatural "affliction" or spiritual attacks. | I used to have no consciousness; I started to fall down. |
| Sudden strangulation | Involuntary movements (like hands moving to the neck) interpreted as spiritual attacks. | I was unaware that I used to strangle my own neck... they brought the traditional healer (*Dhami*). |
| Shivering | Involuntary shaking of the body or head | He started to shiver and I had also been caught by similar kind of sickness. |
